# Can myocardial work help in the therapy of resistant hypertension?

**DOI:** 10.1111/jch.14421

**Published:** 2022-01-09

**Authors:** Marijana Tadic, Cesare Cuspidi

**Affiliations:** ^1^ Klinik für Innere Medizin II Universitätsklinikum Ulm Ulm Germany; ^2^ Medicine and Surgery University of Milano‐Bicocca Milano Italy

In this issue of the Journal, Wang and coworkers used new methods of speckle tracking echocardiography to show the therapeutic effect of sacubitril/valsartan combination on left ventricular (LV) mechanics in hemodialysis patients with resistant hypertension.^1^ Speckle tracking echocardiography provides the set of parameters with better sensitivity and specificity to recognize subtle cardiac changes than conventional echocardiographic parameters.^2^ This particularly refers to LV global longitudinal strain (GLS) that was proven to be a better predictor of survival than LV ejection fraction (LVEF), that was considered as the gold standard for evaluation of LV systolic function for decades.^2^ GLS has many advantages over LVEF that include better reproducibility and outcome prediction. In comparison to Doppler‐derived parameters (pulsed or tissue Doppler), GLS provides angle independent and significantly less load‐dependent measurement.^2^ However, GLS is not completely load independent parameter and study involved hemodialysis patients showed that GLS was different in the same patients before and after dialysis.^3^ Despite this limitation GLS was proven to be an important predictor of cardiovascular (CV) events in hemodialysis patients.^4^

The importance of GLS in arterial hypertension is well established and it has been even involved in the guidelines for imaging in hypertensive patients.^5^ It was also demonstrated that GLS has a significant predictive value, that was higher of LVEF for prediction of CV events.^6^ Hypertensive heart disease, that is frequently developed in patients with resistant hypertension, is characterized by interstitial fibrosis that can be effectively detected by cardiac magnetic resonance and increased extracellular volume, but represents a major challenge for echocardiographic assessment.^7^ Strain evaluation provides the best echocardiographic surrogate for evaluation of interstitial fibrosis not only in hypertension, but also in other diseases that result with interstitial fibrosis. Our group recently summarized findings that showed the correlation between histological myocardial interstitial fibrosis, extracellular volume index and GLS derived by cardiac magnetic resonance, which supports the use of GLS in hypertensive population, as the substitute for interstitial fibrotic changes.^7^

GLS is not a perfect index and, as previously mentioned, it is not completely independent of pre‐ and afterload. Following this important limitation, a new echocardiographic set of parameters called myocardial work (MW), which measures the LV pressure–strain loop analysis using a noninvasive method, has developed. MW incorporates LV pressure and provides additional information to LVEF and strain, which are sensitive to LV afterload. Finally, MW delivers four indexes: global constructive work index, myocardial work index, global myocardial work efficiency, and global wasted work. The loop area represents global MW index and corresponds with the total LV work made from mitral valve closure to mitral valve opening. Global constructive work represents MW during LV shortening in systole and LV lengthening during the isovolumic relaxation phase. Global wasted work demonstrates MW performed during LV lengthening in systole and LV shortening in isovolumic relaxation phase, whereas global work efficiency was calculated according to existing formula.^8^

Wang and coworkers demonstrated that global MW index and global constructive work significantly improved after 12 weeks of therapy. Global MW efficiency improved, while global wasted work reduced after 12‐week treatment, but it did not reach statistical significance.^1^ The authors demonstrated significant and sustained reduction in systolic and diastolic blood pressure (BP) in these patients. The included patients had pronounced LV hypertrophy and significantly increased E/e’ ratio and NT‐proBNP, which would speak in favor of heart failure with preserved ejection fraction (HFpEF). However, it is difficult to conclude in hemodialysis patients.

There are several aspects of this interesting study that deserve further comments. The authors included very limited number of patients (*n* = 18) out of 360 dialysis patients from one center in China, which is why this investigation could serve only as a pilot study on this topic. It is also difficult to anticipate how much these findings would be applicable in larger population.^1^ Additionally, not all patients had preserved LVEF (four out of 18 patients had LVEF < 50%), which affects not only GLS, but also parameters of MW. Sacubitril/valsartan treatment induced borderline increase in GLS, whereas improvement in MW was more prominent (global MW index and global constructive work). This implies better sensitivity of MW parameters to recognize the improvement in LV mechanics in patients with resistant hypertension. Nevertheless, one should consider that MW parameters usually do not have normal distribution and require non‐parametric statistical tests for assessment. In the small study population this can easily lead to false positive results, which is why the current results must be interpretated with caution. There are many possible confounding factors that might influence the improvement of MW parameters in these patients. There are no data about patients’ compliance for the previous antihypertensive therapy and dosage of antihypertensive medications, which could have a significant impact of hemodynamics and LV mechanics. Data show that LV filling pressure, evaluated by E/e’ ratio, did not change over follow‐up period of 12 weeks. Considering the large reduction in BP (22.4 mm Hg for systolic BP and 8.3 mm Hg for diastolic BP),^1^ some improvement in E/e’ ratio would be expected because other studies showed significant improvement in LV diastolic function with lower BP reduction.^9^, ^10^

Even though MW represents a promising tool that should overcome limitations of GLS, it should be underlined that many important points need to be resolved. First, the threshold values for MW parameters are not defined as there are not enough data on this topic. Second, the effect of different risk factors, including hypertension, on MW is not completely resolved because some studies showed positive relationship between systolic BP and global work index and global constructive work ^11^, ^12^ in general and hypertensive population, whereas other studies showed no difference in MW parameters between hypertensive patients at different stage of hypertension.^13^ Recent study demonstrated the significant predictive value of lower cardiac work index on all‐cause mortality in patients receiving regular hemodialysis.^14^ This would imply that hypertensive patients, who typically have higher cardiac work index, are under lower risk for adverse outcomes. These findings underline the importance of longitudinal studies that would investigate long‐term changes of MW parameters in hypertensive population with different BP levels–hypertension stages.

The important question is whether the addition of sacubitril/valsartan combination in patients with resistant hypertension who already receive full antihypertensive therapy was the only responsible for this level of BP reduction. The PARAGON‐HF trial showed that the reduction in systolic BP at weeks 4 and 16, respectively, was greater with sacubitril–valsartan vs. valsartan in patients with resistant hypertension (‐4.8 and –3.9 mm Hg) and mineralocorticoid receptor antagonist (MRA)‐resistant hypertension (‐8.8 and ‐6.3 mm Hg).^15^ The excessive reduction of systolic BP for 22.4 mm Hg might be explained with better compliance of patients for taking other medications due to regular medical visits and check‐up.^1^ Considering the fact that all patients were regularly dialyzed, it is of a great importance when and how BP was measured. The authors explained that BP was measured at home (three times per day), pre‐dialysis and intra‐dialysis. Changes of the home systolic and diastolic BP from the baseline were measured every week. Ambulatory BP measurement was not performed and therefore more detailed information about BP changes are not available.

It should be emphasized that BP has been steeply and continuously reducing over the first 4 weeks of sacubitril/valsartan treatment with plateau between 4th and 8th week, and additional, but significantly smaller, reduction between 8th and 12th week.^1^ The short duration of this study does not allow us to draw the conclusions about the long‐term effect of this therapy in hemodialyzed patients with resistant hypertension. Therefore, it remains unclear if this effect on BP represents maximally achieved BP reduction or perhaps a new plateau followed by an even greater decline in BP. It is also questionable if BP will return on the baseline values after a longer period of follow‐up. Interestingly, the improvement of LV mechanics (GLS and MW) did not proportionally follow this short‐term BP reduction during the first 4 weeks of sacubitril/valsartan therapy when patients reached the major part of effects (approximately 15 mm Hg systolic BP reduction of maximal 22.4 mm Hg achieved after 12 weeks of treatment). The encouraging finding is that only 2/18 patients had symptomatic hypotension, which support safety of prescribing sacubitril/valsartan combination to dialyzed patients with resistant hypertension.^1^

The sample size is very limited to enable statistical analysis that would show if the level of BP reduction correlated with improvement of MW and particularly if this improvement was independent of other parameters such as GLS, E/e’, and LVMI.

MW is feasible and non‐invasive echocardiographic measurement of myocardial performance which includes GLS and effect of afterload (systemic BP). This study shows that MW set of parameters might detect subtle changes in myocardial mechanics that could not be detected even with GLS. Additionally, the present study demonstrated that sacubitril/valsartan therapy is safe and efficient therapy in patients with resistant hypertension who are on regular hemodialysis program. Larger multicenter studies that would include a broad spectrum of patients with arterial hypertension will determine the additional importance of MW parameters on LV performance evaluation and outcomes.

## CONFLICT OF INTEREST

The authors have nothing to disclose.

## AUTHOR CONTRIBUTIONS

Marijana Tadic–writing the commentary paper. Cesare Cuspidi–detailed review with constructive remarks that substantially changed the commentary paper.
